# Ambient particulate air pollution and daily stock market returns and volatility in 47 cities worldwide

**DOI:** 10.1038/s41598-021-88041-w

**Published:** 2021-04-21

**Authors:** Simo-Pekka Kiihamäki, Marko Korhonen, Jouni J. K. Jaakkola

**Affiliations:** 1 Center for Environmental and Respiratory Health Research, Faculty of Medicine, University of Oulu, Oulu, Finland; 2 Department of Economics, Oulu Business School, University of Oulu, Oulu, Finland; 3 Finnish Meteorological Institute, Helsinki, Finland; 4 Biocenter Oulu, University of Oulu, Oulu, Finland

**Keywords:** Climate sciences, Environmental sciences, Environmental social sciences

## Abstract

We studied globally representative data to quantify how daily fine particulate matter (PM_2.5_) concentrations influence both daily stock market returns and volatility. Time-series analysis was applied on 47 city-level environmental and economic datasets and meta-analysis of the city-specific estimates was used to generate a global summary effect estimate. We found that, on average, a 10 μg/m^3^ increase in PM_2.5_ reduces same day returns by 1.2% (regression coefficient: − 0.012, 95% confidence interval: − 0.021, − 0.003) Based on a meta-regression, these associations are stronger in areas where the average PM_2.5_ concentrations are lower, the mean returns are higher, and where the local stock market capitalization is low. Our results suggest that a 10 μg/m^3^ increase in PM_2.5_ exposure increases stock market volatility by 0.2% (regression coefficient 0.002, 95% CI 0.000, 0.004), but the city-specific estimates were heterogeneous. Meta-regression analysis did not explain much of the between-city heterogeneity. Our results provide global evidence that short-term exposure to air pollution both reduces daily stock market returns and increases volatility.

## Introduction

Ambient air pollution has currently dramatic effects on public health and economy worldwide. The World Health Organization estimated that ambient air pollution accounts for 4.2 million deaths per year due to stroke, heart disease, lung cancer and chronic respiratory diseases (https://www.who.int/health-topics/air-pollution#tab=tab_2). Ambient air pollution comprises a complex mixture of components potentially harmful to health, but fine particulate matter (PM_2.5_) is considered to be the driver of health effects. A recent epidemiologic study, conducted in 672 cities or regions in 42 countries worldwide, provides quantitative estimates of the effects of short-term exposure to fine particulate matter on total mortality and mortality from cardiovascular and respiratory diseases^1^. An increase of 10 μg/m^3^ in the 2-day moving average of PM_2.5_ concentration was related to a 0.68% (95% CI 0.59, 0.77) increase in daily all-cause mortality, 0.55% (0.45, 0.66) increase in cardiovascular mortality and 0.74% (0.53, 0.95) increase in respiratory mortality.

Deryugina et al.^2^ assessed overall health effects of PM_2.5_ exposure in the United States and estimated the economic costs of PM_2.5_ exposure. They estimated accounting for age and sex that a 1 μg/m^3^ increase in PM_2.5_ causes the loss of 2.99 life-years per million beneficiaries over 3 days. Using a conventional value of $100,000 per life-year^3^ results in a cost of 299,000 per million beneficiaries. Mortality effects represents a tip of the iceberg in the overall health effects and the overall economic costs are likely to be vast. Thus, many countries have taken action to regulate the air pollution levels, which has led to improvements in air quality, especially in the wealthier countries.

In addition to causing morbidity and mortality, exposure to air pollution is also linked to different types of psychological, economic and social effects^4^. During the last three decades, researchers have tried to assess how different environmental phenomena affect stock prices and returns^5^. A popular and widespread explanation for the potential effects is that certain environmental factors may have a negative effect on the mood of the investors^6,7^. Additionally, there is recent evidence that short term exposure to air pollution can hinder cognitive performance^8^, which could influence the behavior of the investors as well.

The hypothesized relation between air pollution and volatility is more complex than the relation between air pollution and stock returns. Several competing theories have been developed on how environmental factors may influence stock market volatility via the mood pathway, which are discussed by Symeonidis et al.^9^ Worsening of mood (increases in air pollution) can cause more disagreement between the investors and the market valuation, which may then lead to increased volatility^10–12^. Whereas according to other studies, a better mood (decreases in air pollution) may lead to increased trading volumes which would also lead to increased volatility^13,14^. A third theory relies on the assumption that returns and volatility are inversely correlated^15^, and therefore if air pollution decreases returns, it should simultaneously increase the volatility.

In this study we focused on how daily PM_2.5_ concentrations affect stock market returns and volatility. The main focus of the previous literature has been on the associations between daily air pollution concentrations and stock returns^16–23^. Somewhat surprisingly, the relationship between air pollution levels and stock volatility has only been assessed in three research articles^16,19,23^. The results are somewhat controversial; studies focusing on Europe and North America have found that increases in daily air pollution levels decrease stock returns^18–21^, whereas studies with Chinese data have not reported such an association^17,22^. Table 1 summarizes the results, methods and geospatial coverage of the previous studies.

**Table 1:** Characteristics of the previous studies on the relation between daily air pollution levels and stock returns and volatility.

Author(s)	Pub. year	Cities	Study period	Pollutant(s)	Covariates	Methods	Results
Levy and Yagil	2011	New York, Philadelphia	01/1997–06/2007	AQI as binary classification	Monday, January, lag1 returns	OLS, t-test	Significant negative effect for unhealthy AQI days
Levy and Yagil	2013	New York, Philadelphia, Toronto, Amsterdam, Sydney, Hong Kong	01/1997–06/2007	AQI	lag1 returns, Monday, full moon, fog, season	OLS	Significant negative effect for an increase in AQI
Lepori	2016	Milan	01/1989-05/2006	PM_10_, lag1 and 3dMA	lag1-2 returns, SAD, fall, fullmoon, newmoon, Monday, tax, temperature, rain, month	Binary logit	Significant negative effect on centralized market ren1rns. Stronger effects for 3dMA. Found the effects for NO_x_ and SO_2_ as well
Li and Peng	2016	Shanghai, Shenzhen	01/2005–12/2014	AQI	Humidity, wind speed, SAD, Monday, January, lag1 returns, lags 1-2 for AQI	OLS	Significant negative effect only for time period 2010–2014. Noticed a rebound effect with lag2 AQI
Heyes et al.	2016	New York	01/2000–11/2014	PM_2.5_	lagl-2 returns, temperature, dew point, precipitation, wind speed, air pressure, cloud cover, O_3_, CO, day of the week, tax dummy, year-by-week	OLS	Significant negative effect (− 0.0171) for a unit increase of PM_2.5_. Various additional sensitivity and robustness checks
He and Liu	2018	Shanghai	02/2005–03/2017	AQI	Temperature, humidity, wind speed, precipitation, cloud cover, SAD, Monday, January, lag1 return	OLS	Found no relationship between air quality and the stock returns
Wu et al.	2018	33 Chinese cities	12/2013–12/2015	AQI, PM_2.5_, PM_10_,SO_2_, CO, NO_2_,O_3_	lag1 returns, SAD, humidity, temperature, air pressure, visibility, wind speed, cloud cover, 30-day average return, Monday, month	Cross- sectional panel data regression	Observed a significant negative effect between air pollution and stock returns
An et al.	2018	Chinese national aggregate	01/2014–12/2015	AQI	Investor sentiment	OLS, GARCH	Found a statistically significant relationship between air quality and returns, but not between air quality and volatility

Furthermore, there are not enough studies to generalize the results globally or to explain the differences in associations between market locations. Our aim was to fill this gap in knowledge by using data from 47 stock exchange cities around the globe. We tested the following hypotheses:

### Hypothesis 1

Increases in the levels of daily PM_2.5_ concentrations decrease daily stock returns.

### Hypothesis 2

Increases in the levels of daily PM_2.5_ concentrations increase daily stock volatility.

We applied regression models for each city individually. The volatilities of the stock indices were modeled with GJR-GARCH(1,1) method, which has been developed for accounting for asymmetric shocks^24^. In addition to traditional time-series analysis methods, we applied random effects meta-analysis^25^, which has been rarely used in economics but more commonly in environmental epidemiology^1,26^, to summarize the global effects of air pollution on stock returns and volatility.

## Results and discussion

### Associations between daily PM_2.5_ concentrations and stock returns

**Figure 1:**
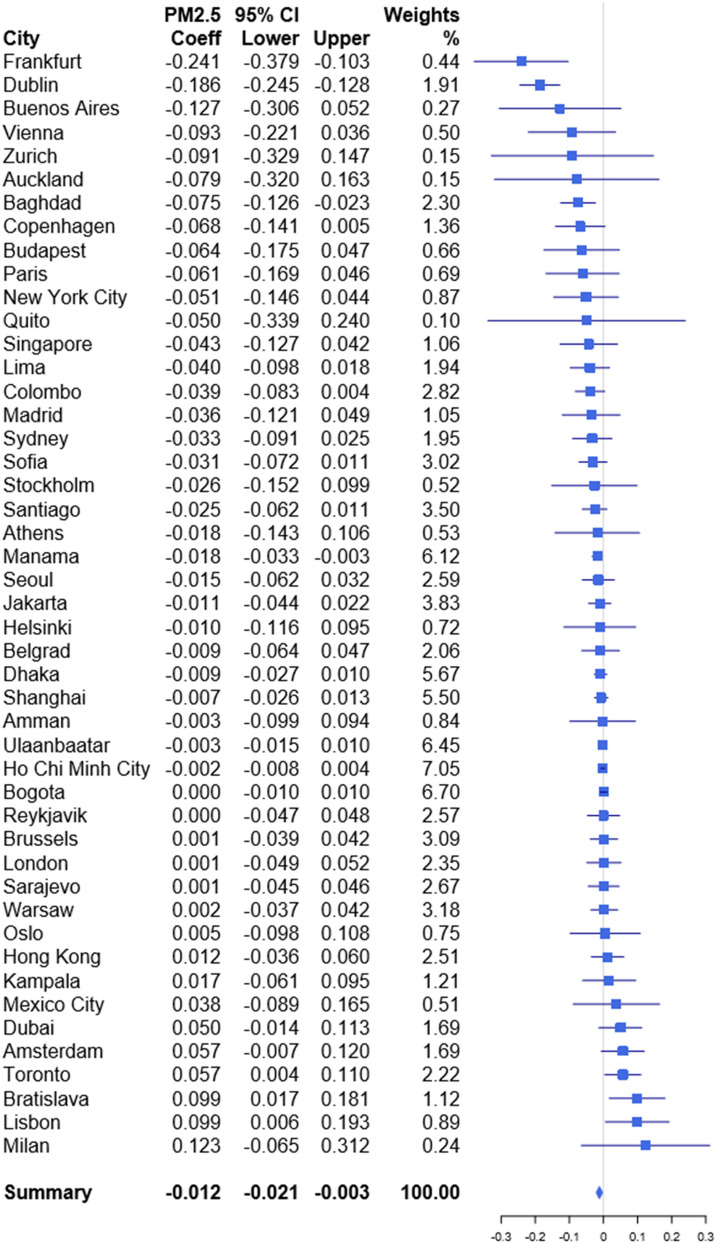
Regression coefficients and 95% confidence intervals for the association between 10 μg/m^3^ increase in daily PM_2.5_ concentration and stock index returns.

Our main results are presented in Fig. 1, where the effect estimates are based on Eq. (2) presented in “Methods” section. The effect estimates, and their 95% confidence intervals, are presented for each city individually. The results are in line with our hypothesis that investors exposure to PM_2.5_ would lead to decreased stock returns. The summary effect estimate for PM_2.5_ based on 47 city specific estimates is − 0.012 (95% confidence interval − 0.021, − 0.003), which is statistically significant at the 5% level. The effect estimate corresponds to an average 1.2% reduction in stock returns per a 10 μg/m^3^ increase in PM_2.5_. A rather substantial amount of heterogeneity can be observed when comparing the results of individual cities (I^2^ = 48.64%).

We quantified the effects with different lags (see Appendix figures A2–A5) and found that the effect persisted when applying a 2-day moving average. Epidemiological research suggests that air pollutants have distributed lag effects on health over subsequent days^27^ which is also backed by the results of Lepori et al.^19^ who found that the association was stronger when applying a 3-day moving average instead of the index day concentration. In our sample, the 2-day and 3-day moving averages provided similar summary effect estimates, 2-day: − 0.009 (− 0.018, − 0.000); 3-day: − 0.012 (− 0.025, 0.001), as the index day with some differences in individual cities. The other lagged patterns did not produce significant results, which implies that the PM_2.5_ effect on investors has a rather short induction period. In addition to the shorter lags, we tested the individual cities for a 30 lag-day cumulative association. The long-term cumulative effects were not significant across the board.

Besides the overall pooled effect estimate, we present all the city-specific effect estimates which are somewhat similar to the corresponding estimates observed in the previous studies. Similarly to both Li and Peng^22^ and He and Liu^17^ we did not observe any significant effect in Shanghai. However, in our case, the effect estimate is of similar size as the pooled effect; − 0.007 (95% confidence interval: − 0.026, 0.013). Lepori^19^ used a 3-day average PM_10_ concentration as a predictor, and observed that the Italian stock market returns were 2% less likely to be positive when PM_10_ levels rose by 10 μg/m^3^. While not directly comparable, our estimate has a positive sign, which implies that returns in Milan have been higher on days with higher PM_2.5_. levels. The estimate, however, is not statistically significant which could be related to a small sample size; our sample for Milan only covers a short 1-year time span.

Finally, if we compare the present results from New York City, − 0.051 (− 0.146, 0.044), with those from Heyes et al.^18^ who estimated a coefficient of − 0.0171 for 1 μg/m^3^, we can notice some difference. The previous studies^20,21^ also found a statistically significant relationship in New York, whereas we did not. In fact, the point estimate presented by Heyes is over 3 times larger than ours. This is a slight surprise, since we used highly similar methods and data as was used in the article by Heyes et al.^18^. The main difference between the analyses, that we could think of, is the time span. Their study period reaches from 2000 up to 2014, while our data sample ranges between 2007 and 2019.

In order to understand the variation of the effects over time, we split the data into four five-year strata and analyzed them as shorter periods (Appendix figures A6–A9). Due to most of the data being from the last five years, we believe that assessing the summary estimates for each period is not meaningful. However, we observed some interesting patterns that further underlines that studying the phenomenon with a large geopgraphical and temporal scope is important. For example, the effect estimates for Athens and Helsinki are close to 0 and statistically insignificant when using the full data sample, whereas these cities have a highly significant negative coefficient in the 2011–2015 and 2016–2019 samples respectively.

We studied also the role of traffic emissions as potential confounders of the association between PM_2.5_ and stock returns. In addition to PM_2.5_, traffic emissions include other pollutants. We did not have direct information on traffic emissions or volumes, but we used NO_2_ as an indicator of traffic emissions in the European cities. Inclusion of NO_2_ in the models did not influence the pooled effect estimates (see Appendix figures A10 and A11). The summary effect estimate for the European cities was 25% stronger than in the full sample.

### Associations between daily PM_2.5_ concentrations and volatility

**Figure 2:**
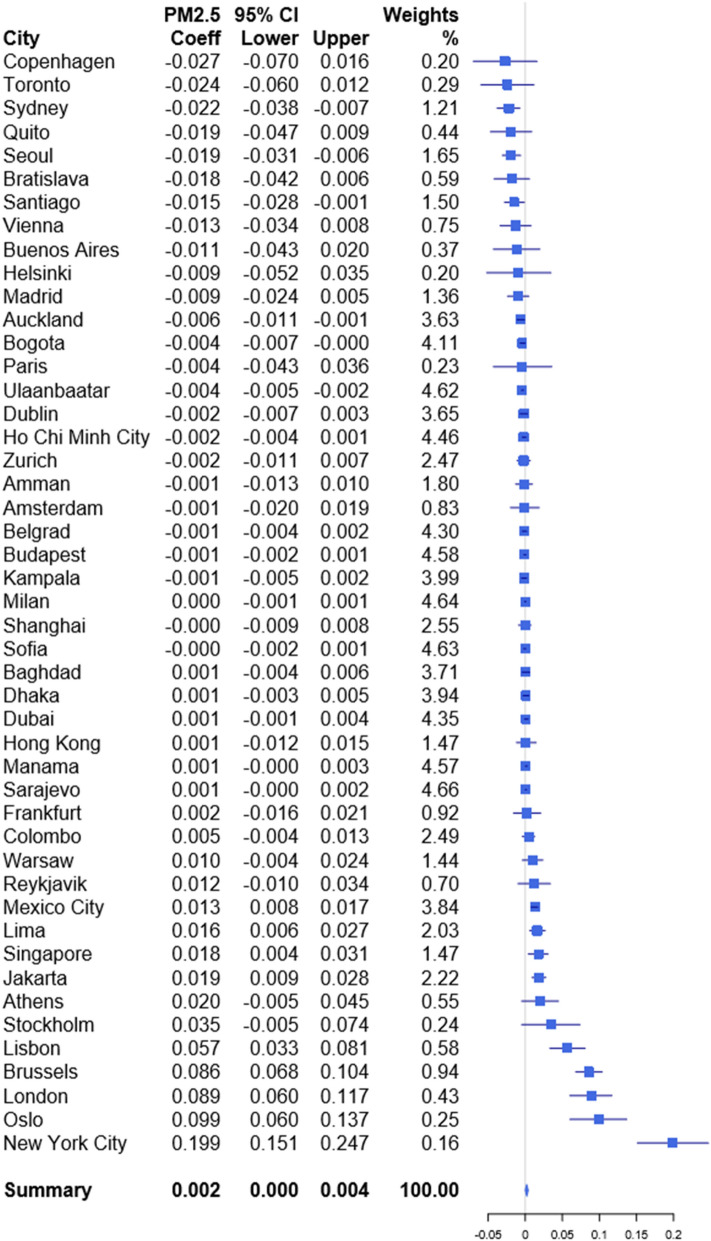
Regression coefficients and 95% confidence intervals for the association between 10 μg/m^3^ increase in daily PM_2.5_ concentration and stock index volatility.

The results for the volatility models are presented in Fig. 2. The summary effect estimate (0.002 per 10 μg/m^3^increase in PM_2.5_, 95% CI 0.000,0.004) indicated that PM_2.5_ concentration increases the stock market volatility, but there was substantial heterogeneity between the city-specific effect estimates (I^2^ = 88.22%).From the GJR-GARCH parameter estimates we found that the leverage term γ was positive (36 out of 47) with no statistical significance (35 out of 47) in most of the locations (See Appendix Table A2). Only Ulaanbaatar had a statistically significant negative γ term. This means that, in the majority of the locations, negative shocks have more impact on volatility than positive shocks^28^.

The effect estimate for New York City was the largest at 0.199 (0.151, 0.247) which represents a rather substantial 22% increase in volatility when the PM_2.5_ rose by 10 μg/m^3^. The magnitude of the estimate for New York City was much larger than those of any other cities. The difference could not be attributed to geographical areas as the other North American city, Toronto, ended up with the second highest negative estimate. Additionally, there were four European cities (Oslo, London, Brussels, and Lisbon) where the effect estimate was considerably large and statistically significant at the 95% level.

The observed heterogeneity is not that surprising. In fact, Symeonidis et al.^9^ used a similar approach to model the effects of weather conditions on stock market volatility using a cross-sectional data that contained multiple cities as well. Their results have, similarly, a wide amount of variance between the results of individual cities. One possible explanation is that other underlying features related to the population could modify the effects of environmental factors on investor behavior. For example, the level of internationalization of the stock market, reliance on technology (such as algorithmic trading), or cultural features of the affected traders, could influence how the decreased mood or risk-aversion manifests on the stock market.

We adjusted for traffic emissions as a potential confounder also for the association between PM_2.5_ and stock market volatility by adding NO_2_ as an indicator of traffic-related emissions into the model (see Appendix figures A12 and A13). Interestingly, the summary effect estimate for the European cities decreased from 0.008 (95% CI 0.003, 0.012) in the unadjusted model to 0.004 (95% CI 0.000, 0.008) in the model adjusting for NO_2_. These findings indicate some confounding by NO_2_, but show an independent effect of PM_2.5_. It is also worth noting that the effect estimate for Europe only is significantly higher than the global summary effect estimate.

### Determinants of between-city heterogeneity in effect estimates for returns and volatility

We observed significant between-city variability in the effect estimates of our main regression analyses. The physiological effects of PM_2.5_ exposure on humans should be consistent everywhere. We believe that some other type of phenomena might explain the differences in the city-specific results. An easy explanation would be, that the PM_2.5_ measurements do not describe accurately enough the exposure on those who are trading the stocks. For example, internet has made reaching the various stock markets from farther distances relatively easy and thus the proportion of international traders might be large enough to offset any effects of local air pollution concentrations. Furthermore, the amount of international trading at the stock markets may vary strongly, and thus be a significant source of the observed heterogeneity. Unfortunately, we were unable to attain relevant data for quantifying the phenomenon, or to locate where the majority of the traders participate the stock trading from.

In order to explain the differences in the city-specific estimates, we applied meta-regression on the results. The key explanatory variables tested for these analyses were study period (start year, end year, length of time span), the mean, range and interquartile-range of PM_2.5_ concentration and daily returns, market capitalization of the stock market, and geographic location based on continents, and Köppen climate classification. The market capitalization was dichotomized as:

 < 100 billion = 0.

 >  = 100 billion = 1.

The limited number of cities and rather large deviance between the city-specific regression estimates creates some issues for the meta-regression. The relatively small number of independent observations (N = 47) limits the power of the analyses. Similarly, the available degrees of freedom within the model does not allow fitting many explanatory variables simultaneously.

The best fitting meta-regression model for the returns models (I^2^ of 54.16%) included mean returns, mean PM_2.5_ concentrations (μg/m^3^), and the market cap dummy as predictors. The mean concentration of PM_2.5_ provided an estimate of 0.0005 (95% CI 0.0000, 0.0010) per a 10 μg/m^3^ increase in PM_2.5_, which would suggest that the effect of PM_2.5_ on daily returns is stronger in locations where the concentrations are lower on average. If we assume that the exposure–response function for the phenomenon is S shaped, it would be plausible that a short-term increase in PM_2.5_ concentrations would have a smaller effect in locations where the average concentrations are already high.

Furthermore, the meta-regression estimate for the mean daily return was also significant with a coefficient of − 0.4928 (− 0.7832, − 0.2024), which would suggest that the effect is stronger in markets where the returns are higher on average. The estimate for the market cap dummy was not significant even at 10% level, but the coefficient 0.0170 (− 0.0066, 0.0407) suggests that the negative effect of air pollution might be stronger in locations were the market capitalization is smaller. This could indicate that the level on internationalization of the smaller stock exchanges is also smaller, and therefore the effect of local air pollution would also be stronger.

We attempted to apply meta-regression on the volatility models as well. However, our predictors did not seem to catch much of the heterogeneity. The I^2^ statistic remained consistently at very high levels. The best fitting model (I^2^ = 88.22%) included only the mean daily returns with an estimate of − 0.0440 (− 0.0855, − 0.0025). The results imply that the PM_2.5_ effect on volatility is lower in stock exchanges where the returns are higher on average.

The previous studies have focused mainly on individual cities, and at most only tested the models in a couple of additional locations. In this study we have presented that the effects vary between time and space, which was also noted by Li and Peng^22^. Focusing on a single or a handful of cities may provide results that are only relevant in the studied location and only during the specific time span of the study. Therefore, generalizing or drawing conclusions from such results can be misleading. By using long temporal and wide geospatial coverage we increased the credibility of the results and provided evidence that the studied associations between daily PM_2.5_ concentrations and stock returns/volatility exist on a global scale, even if their observed magnitude may vary between time and place.

## Conclusions

This study presents a major contribution to economics by expanding the geographic coverage of the studies related to the association between air pollution exposure and stock market returns and volatility. We believe that these findings are also of significant importance in environmental health on providing indirect evidence that short-term exposure to air pollution can have substantial effects on mood and human cognitive functions, which in turn affects investor behavior.

The results of our global assessment on the effects of PM_2.5_ on stock market returns provide evidence that on average, a 10 μg/m^3^ increase PM_2.5_ concentration decreases the daily returns by 1.2%. Furthermore, we show that these associations are stronger in areas where the average PM_2.5_ concentrations are lower, the mean returns are higher, and where the local stock market capitalization is low.

Additionally, we present evidence that increases in the PM_2.5_ levels also influence the stock market volatility. The latter results are notably heterogenous and can not be generalized in a global scale. Regardless of the lack of generalizability, the findings further strengthen the hypothesis that air pollution influences investors' behavior.

Additional research on the causal mechanisms is still needed as we do not yet know why the investor behavior changes along variations in the levels of air pollution. The current study could also be expanded to cover additional stock market related responses such as trading volumes, and a wider set of factors for explaining the observed heterogeneity.

## Methods

### Data

Our aim was to assess globally the effects of air pollution on stock markets. There are 144 stock exchanges worldwide. Investing.com, to our knowledge, has the largest inventory of the exchanges and the indices traded in each of them. In order to get a global geospatial coverage for the study, we searched their locations and filtered out all major indices. This generated a list of 88 cities representing the stock market capitals of their respective regions.

**Figure 3:**
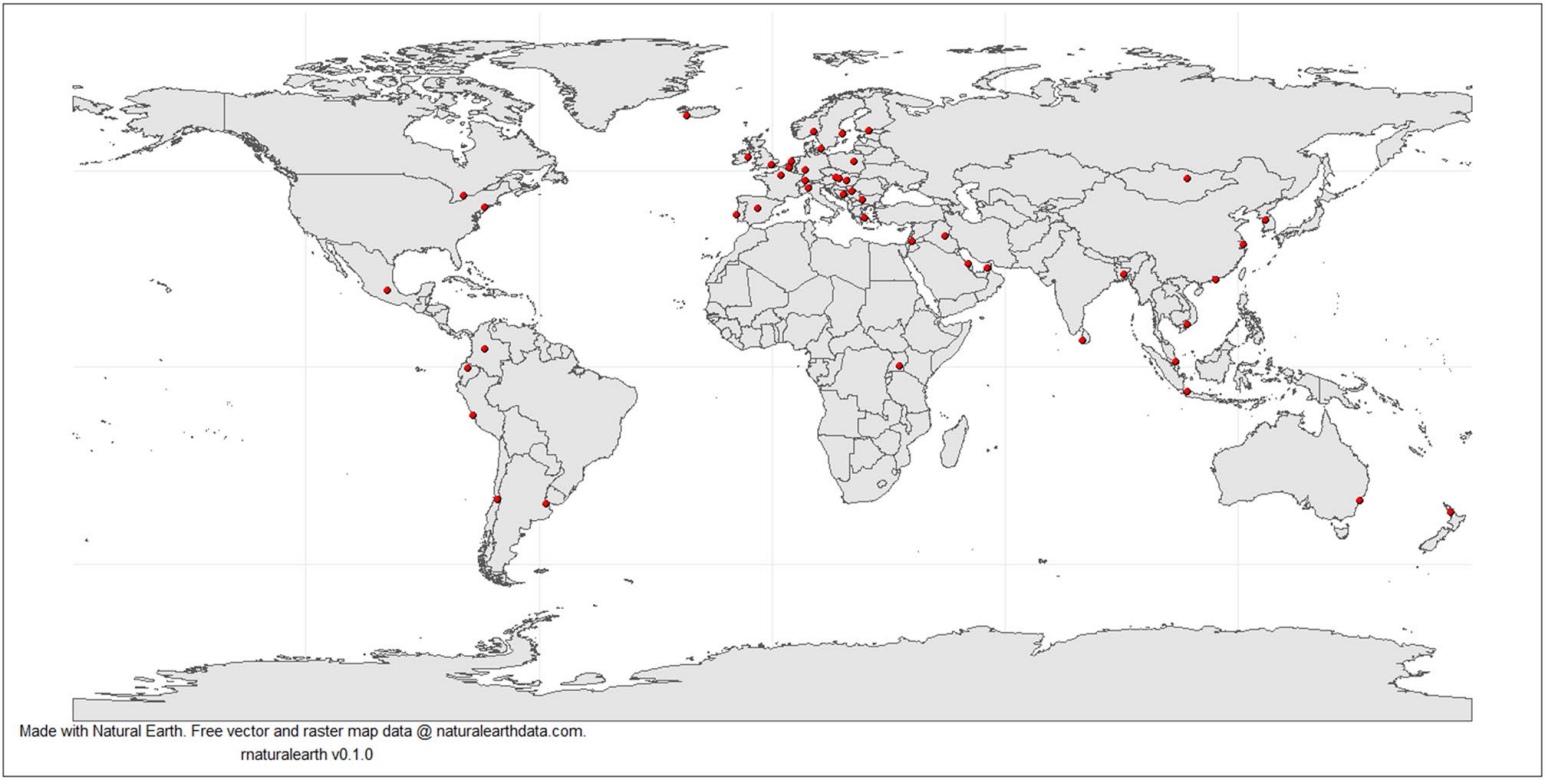
Geographical coverage of the study.

After attaining a wide range of possible cities, we identified the coordinate points of the stock exchanges and searched for air quality data from as close to the coordinate point as possible. We found daily air pollutant concentration measurements for 54 of the 88 cities in total. Out of these cities, 47 contained a sufficient amount of PM_2.5_ measurements to be included in the analysis. The geospatial coverage of the study is illustrated in Fig. 3. The data sources for all air quality monitoring stations are provided in the supplementary material (Metadata spreadsheet). Most of the cities where air quality measurements could not be found were located in Asia and Africa. Finally, we downloaded matching meteorological data for the respective locations from the NOAA GSOD database (https://data.noaa.gov/dataset/dataset/global-surface-summary-of-the-day-gsod).

Since we cannot assess the empirical relation between stock returns and PM_2.5_ on the days they were not measured, we omitted all days where either one of the values was missing. The total number of observations was 47 928, the time spans for each city ranged between 1 and 19 years and the median time span was 4 years. Table 2 presents the mean (sd) for the main interest variables daily returns and PM_2.5_ concentrations, as well as the time span covered in the data.

**Table 2:** Characteristics of the 47 stock market cities included in the present study.

City	Study period	Returns, mean (sd)	PM_2.5_, mean (sd)
Amman	2019–2019	− 0.028 (0.213)	23.273 (6.181)
Amsterdam	2013–2018	0.011 (0.976)	14.549 (9.434)
Athens	2016–2019	0.076 (1.300)	19.174 (9.414)
Auckland	2017–2019	0.088 (0.549)	6.869 (1.990)
Baghdad	2019–2019	− 0.004 (0.460)	36.807 (18.471)
Belgrad	2016–2019	0.052 (0.535)	23.209 (18.957)
Bogota	2016–2019	0.035 (0.724)	17.789 (28.483)
Bratislava	2017–2019	0.038 (0.946)	16.089 (10.675)
Brussels	2006–2019	0.008 (1.230)	21.264 (13.904)
Budapest	2019–2019	0.055 (0.743)	14.006 (9.354)
Buenos Aires	2015–2019	0.192 (2.098)	16.138 (9.961)
Colombo	2017–2019	− 0.014 (0.419)	28.368 (14.391)
Copenhagen	2004–2018	0.012 (1.291)	13.280 (6.346)
Dhaka	2016–2019	0.053 (0.601)	77.646 (56.423)
Dubai	2018–2019	0.037 (0.758)	48.351 (26.022)
Dublin	2019–2019	0.143 (0.878)	10.086 (8.102)
Frankfurt	2017–2019	0.034 (0.858)	9.838 (6.194)
Helsinki	2005–2017	0.021 (1.415)	9.523 (5.704)
Ho Chi Minh City	2016–2019	0.070 (0.934)	41.448 (68.312)
Hong Kong	2011–2019	− 0.008 (1.115)	27.096 (14.899)
Jakarta	2015–2019	0.026 (0.779)	44.702 (17.018)
Kampala	2017–2019	0.042 (0.796)	57.362 (18.132)
Lima	2016–2019	0.040 (0.757)	33.268 (13.482)
Lisbon	2010–2019	− 0.041 (1.199)	13.325 (7.470)
London	2001–2019	0.013 (1.145)	13.790 (8.310)
Madrid	2004–2005	0.048 (0.690)	21.385 (9.579)
Manama	2016–2019	0.026 (0.414)	57.305 (25.026)
Mexico City	2012–2014	− 0.018 (0.818)	29.528 (9.250)
Milan	2018–2018	− 0.062 (1.059)	21.019 (13.142)
New York City	2007–2019	0.023 (1.190)	9.568 (5.681)
Oslo	2010–2019	0.055 (1.186)	9.982 (6.273)
Paris	2011–2018	0.042 (1.212)	14.784 (11.747)
Quito	2016–2019	0.059 (0.458)	17.219 (5.099)
Reykjavik	2006–2019	0.004 (0.962)	7.496 (9.020)
Santiago	2009–2019	0.039 (0.861)	27.782 (14.417)
Sarajevo	2016–2019	− 0.018 (0.723)	27.657 (21.552)
Seoul	2015–2019	0.005 (0.770)	23.301 (13.176)
Shanghai	2011–2017	0.033 (1.421)	47.740 (40.938)
Singapore	2016–2019	0.021 (0.713)	16.043 (7.527)
Sofia	2019–2019	− 0.083 (0.453)	19.906 (18.548)
Stockholm	2004–2007	0.028 (0.976)	14.216 (6.587)
Sydney	2015–2019	0.018 (0.814)	7.278 (7.113)
Toronto	2004–2017	0.017 (1.109)	7.458 (6.227)
Ulaanbaatar	2015–2019	0.036 (1.057)	96.620 (116.081)
Warsaw	2009–2019	− 0.023 (1.164)	28.810 (16.801)
Vienna	2017–2019	0.039 (0.896)	14.844 (11.220)
Zurich	2019–2019	0.111 (0.592)	9.041 (6.081)

### Imputation

**Figure 4:**
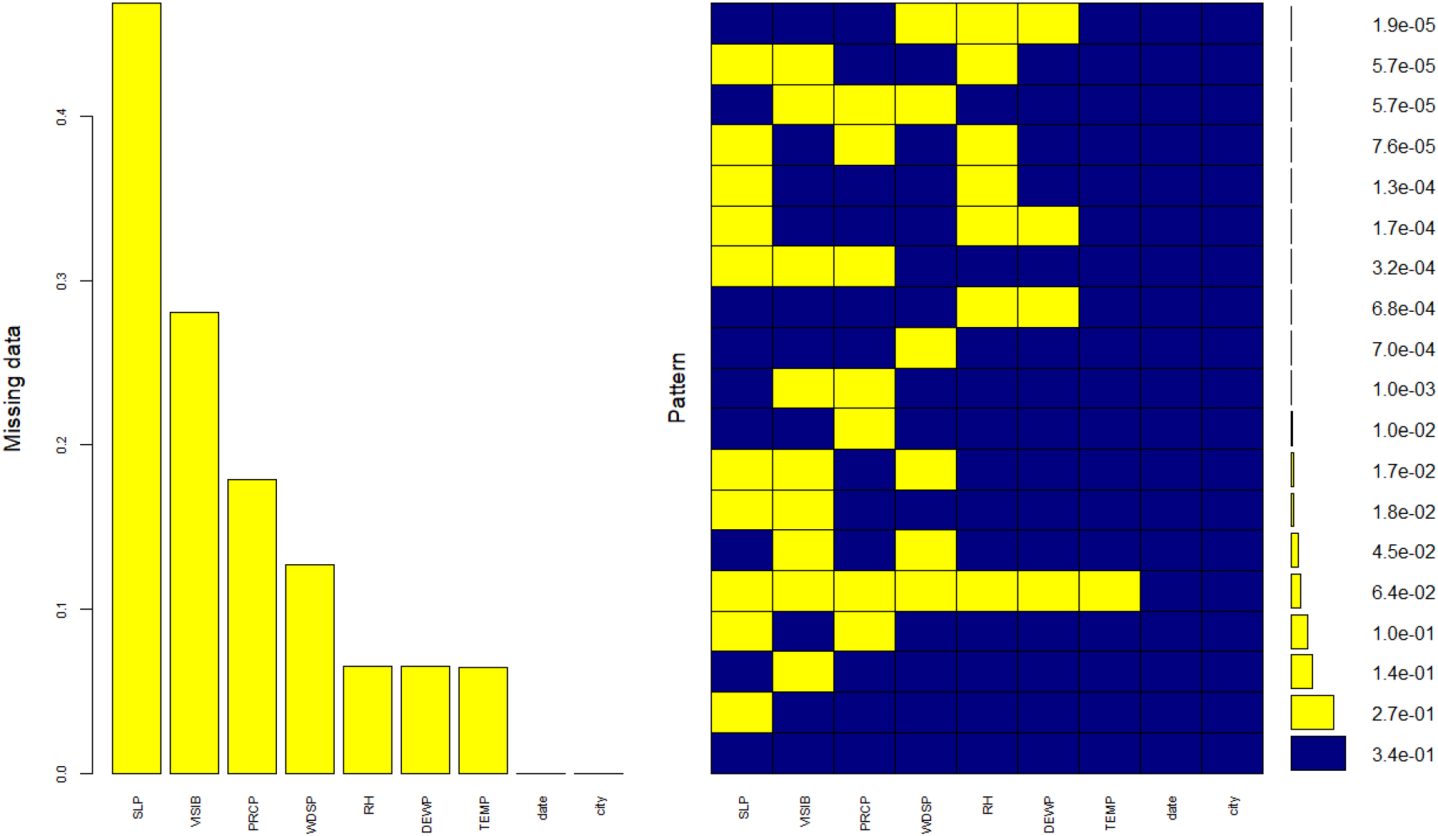
Patterns of missing data.

A preliminary investigation revealed that the meteorological variables contained a significant amount of missing data, which can be seen from Fig. 4. Most of the missing data were seemingly at random. We, however, identified also larger sections of consecutive missing values. Apparently, certain weather parameters of interest had not been measured in all cities during the study periods. In order to assess the research question while accounting for the weather-related confounding effects, we decided to impute the missing values in order to keep the number of observations at good levels. We assumed that the meteorological phenomena were non-linearly correlated with each other. Therefore, we performed a multiple imputation using the CART (Classification and Regression Training) method from MICE package for R.

### Model and feature selection

Based on previous literature, we identified a wide set of variables that could be used in the regression models. First, we tried several different ways of capturing temporal variations in the data. Similarly to Heyes et al.^18^, we found that including week of year and day of week dummies was an effective method for accounting for non-linearities in the temporal trends. In addition, we followed a common practice in stock-related predictions and included 1- and 2-day lagged returns as predictors to account for autocorrelation in the dependent variable.

We defined stock returns as: $RET_{t} = (\ln \left( {P_{t} } \right) - \ln (P_{t - 1} ))*100$, where P_t_ is the closing price of the city’s corresponding stock index on day t. Then, we formulated the following baseline (1) model:

1 $$RET_{t} = \beta_{0} + \beta_{1} PM_{{2.5_{t} }} + \beta_{2} RET_{t - 1} + \beta_{3} RET_{t - 2} + \beta_{4} woy_{t} + \beta_{5} dow_{t}$$

where $RET_{t}$ denotes the daily return on day t and is defined as, PM_2.5_ the daily concentration, and woy and dow representing the week of year and day of week dummies respectively.

To systematically elaborate on the role of the remaining set of variables, we formed a grid containing each combination of them. Then we added each combination together with the fixed baseline model and iterated an OLS regression for each city with each combination. This approach proved to be efficient for testing the effects and importance of each individual variable and it also served as a test of sensitivity for the relation of main interest. Some variables, such as relative humidity and lunar cycle indicators, were excluded since they did not improve the model and had significant collinearity with other included variables. Furthermore, we explored the lagged associations of PM_2.5_ and the stock returns and concluded, that the effect was strongest when using the index day values.

### Final model specifications

The final model (2) was then constructed based on both earlier literature and rigorous experimentation with the data. The final model:

2 $$\begin{gathered} RET_{t} = \beta_{0} + \beta_{1} PM_{{2.5_{t} }} + \beta_{2} RET_{t - 1} + \beta_{3} RET_{t - 2} + \beta_{4} woy_{t} + \beta_{5} dow_{t} + \beta_{6} PRCP_{t} \hfill \\ + \beta_{7} WDSP_{t} + \beta_{8} SLP_{t} + \beta_{9} VISIB_{t} + \beta_{10} TEMP\_bins_{t} + \beta_{11} DEWP\_bins_{t} + \beta_{12} SAD + \varepsilon_{t} , \hfill \\ \end{gathered}$$

where PRCP, WDSP, SLP, and VISIB denote precipitation, wind sed, sea level pressure, and visibility respectively. These meteorological variables were treated as continuous. *SAD* denotes the daylength in hours, and is treated as a continuous variable in the models. Temperature (TEMP_bins) and dew point (DEWP_bins) were treated as dummy bins, each bin having a width of 2.5 °C to account for non-linearities, a method adopted from the article by Heyes et al.^18^. Finally, the Newey-West standard errors ε_t_ were applied to adjust for arbitrary serial correlation within the models. In the sensitivity analyses of the European cities, we assessed potential confounding by traffic emissions by fitting daily NO_2_ concentrations into the models.

### gjrGARCH volatility model

Next, we moved on to test the effects of air pollution on stock market volatility. For this analysis, we implemented a 3-step GJR-GARCH regression in order to accurately define the volatility and to explain how the environmental factors influence it. First, in order to account for the independent effects in the mean model, we took the residuals from the final returns model (2). Second, we applied a GJR-GARCH(1,1) model on the residuals to model the stock volatilities. Third, once we had obtained the volatilities, we performed an OLS regression using the volatilities as the dependent variable.

The volatility can then be defined as follows:

$$\sigma_{t}^{2} = \omega + \left( {\alpha + \gamma I_{t - 1} } \right)\varepsilon_{t - 1}^{2} - 1 + \beta \sigma_{t - 1}^{2} ,$$

where the ${\text{I}}_{t - 1}$ indicator function is:

$${\text{I}}_{t - 1} \left( {{\upvarepsilon }_{t - 1} } \right) = {\upvarepsilon }_{t - 1} for {\upvarepsilon }_{t - 1} > 0\,\,\,\, and\,\,\,{\text{I}}_{t - 1} \left( {{\upvarepsilon }_{t - 1} } \right) = 0{\text{ otherwise}}$$

A GJR type of GARCH model was selected because it can account for asymmetric nature of the volatility^24^ and can be flexibly fit on different datasets. It has also been used for similar studies by, for example, Symeonidis et al.^9^ and Dowling and Lucey^29^.

### Global estimation via meta-analysis

In order to capture the global effects, we used a similar approach as Liu et al.^1^ who studied the effects of particulate matter on mortality in 652 cities. Essentially, the regression models were performed individually for each city, and finally summarized using a random effects meta-analysis^25^. In this manner, we were able to account for heterogeneity between the cities. Finally, we interpret the summary-effect estimate as a generalized global average effect. We chose this method over panel-regression since we expect there to be significant amount of between-city heterogeneity. A panel regression model assumes that the temporal and meteorological factors have equal effects in different cities. We argue that by allowing the confounders and temporal trends to flexibly adjust individually for each city, the results for the effect of main interest will be more valid. In the sensitivity analyses, we elaborated the role of traffic emissions as a potential confounder by fitting NO_2_ concentrations in the models.

## Supplementary Information

Supplementary Information

## Data Availability

The imputed data set for replicating the results presented in the article is available at Mendeley [10.17632/z8t3s8btxv.1]. Additionally, the sources of data for each individual city are described in the included metadata file.

## Code availability

Code for the analyses is available from Mendeley [10.17632/z8t3s8btxv.1] along with the data.
