# Supplementary material for: Ambient particulate air pollution and daily stock market returns and volatility in 47 cities worldwide
Source: Sci Rep. 2021 Apr 21;11:8628. doi: 10.1038/s41598-021-88041-w (PMC8060286; doi:10.1038/s41598-021-88041-w)
Supplement: Supplementary file 1 — Supplementary Information [file 41598_2021_88041_MOESM1_ESM.docx]

**Ambient particulate air pollution and daily stock market returns and volatility in 47 cities worldwide**

Authors

Simo-Pekka Kiihamäki^1^, Marko Korhonen^2^, Jouni J.K. Jaakkola^1,3,4^

Affiliations

**1. Center for Environmental and Respiratory Health Research, Faculty of Medicine, University of Oulu, Oulu, Finland**

**2. Department of Economics, Oulu Business School, University of Oulu, Oulu, Finland**

**3. Finnish Meteorological Institute, Helsinki, Finland**

**4. Biocenter Oulu, University of Oulu, Oulu, Finland**

Contributions

Study design (S.P.K, M.K, J.J.K.J); data collection (S.P.K); data analysis (S.P.K, M.K); writing of manuscript (S.P.K); data interpretation and editing of manuscript (S.P.K, M.K, J.J.K.J); comments and discussion on the manuscript (M.K, J.J.K.J).

Corresponding author

Jouni J.K. Jaakkola

Correspondence to jouni.jaakkola@oulu.fi

**For Online Publication**

**A Figure 1.** Regression coefficients and confidence intervals for the association between daily PM2.5 concentration and stock index returns. Baseline model (1).
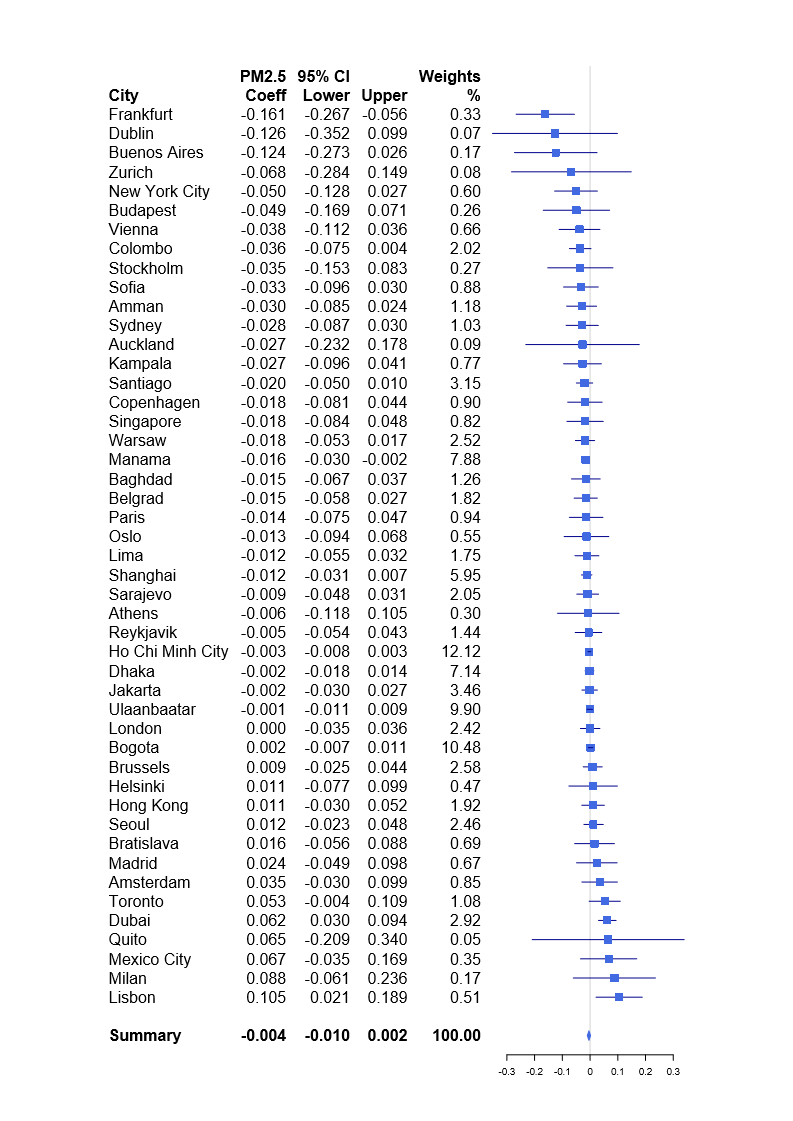


**A Figure 2.** Regression coefficients and confidence intervals for the association between 1-day lagged PM2.5 concentration and stock index returns. Full model (2).
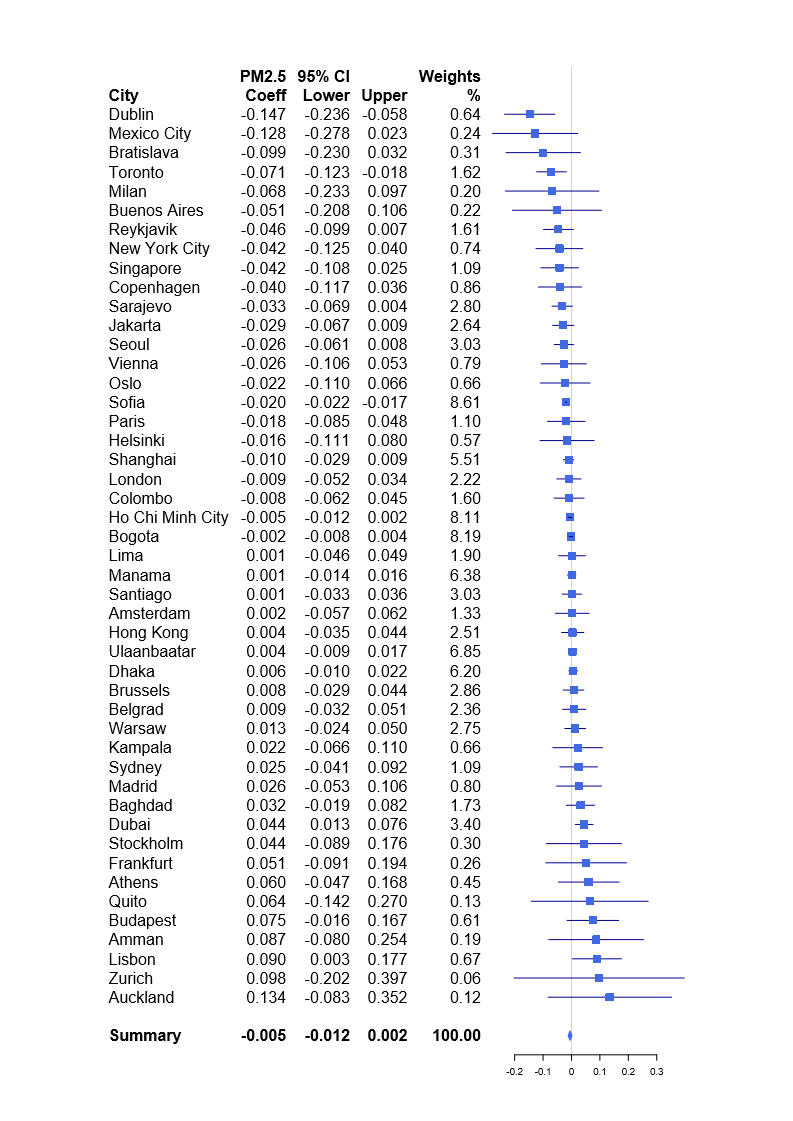


**A Figure 3.** Regression coefficients and confidence intervals for the association between 2-day lagged PM2.5 concentration and stock index returns. Full model (2).
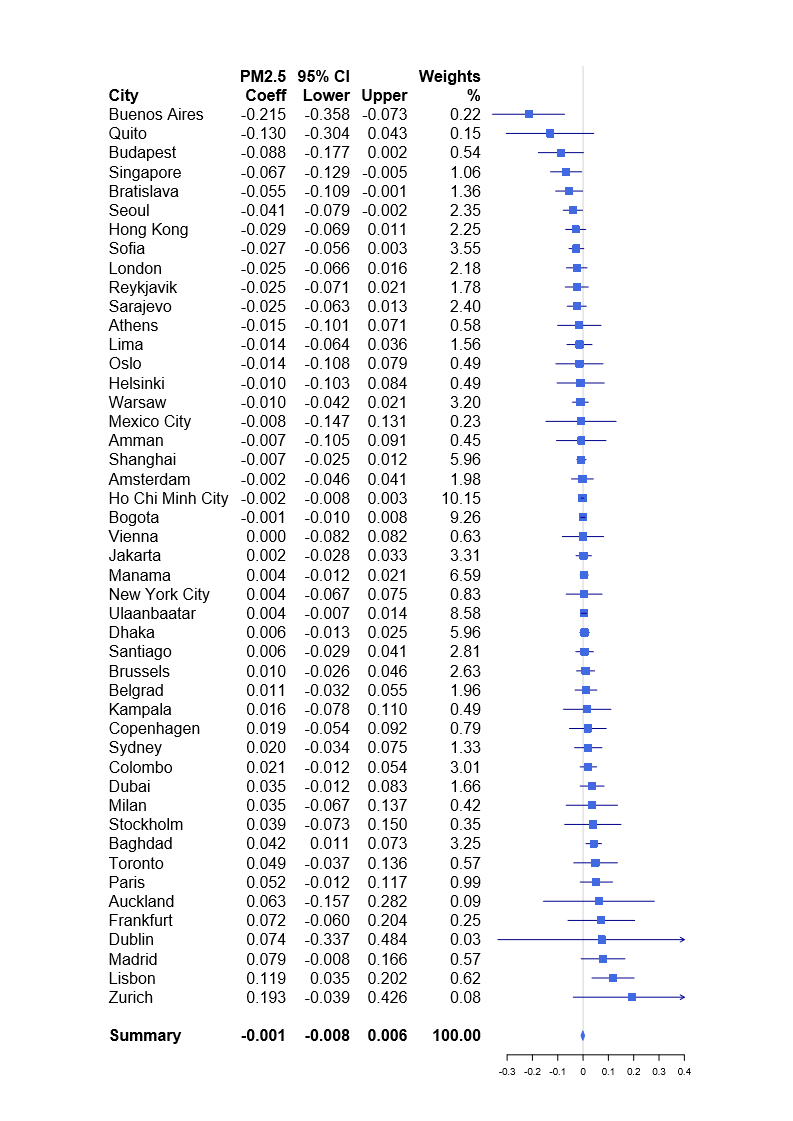


**A Figure 4.** Regression coefficients and confidence intervals for the association between 2-day mean PM2.5 concentration and stock index returns. Full model (2).
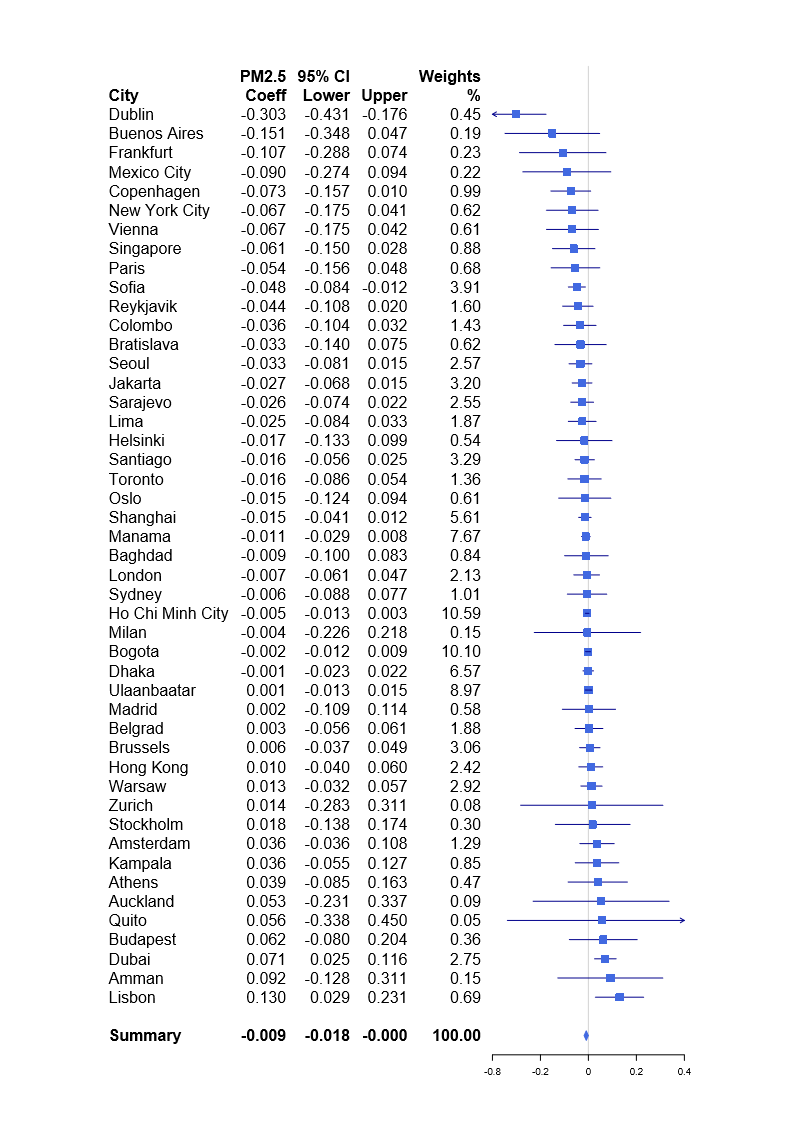


**A Figure 5.** Regression coefficients and confidence intervals for the association between 3-day mean PM2.5 concentration and stock index returns. Full model (2).
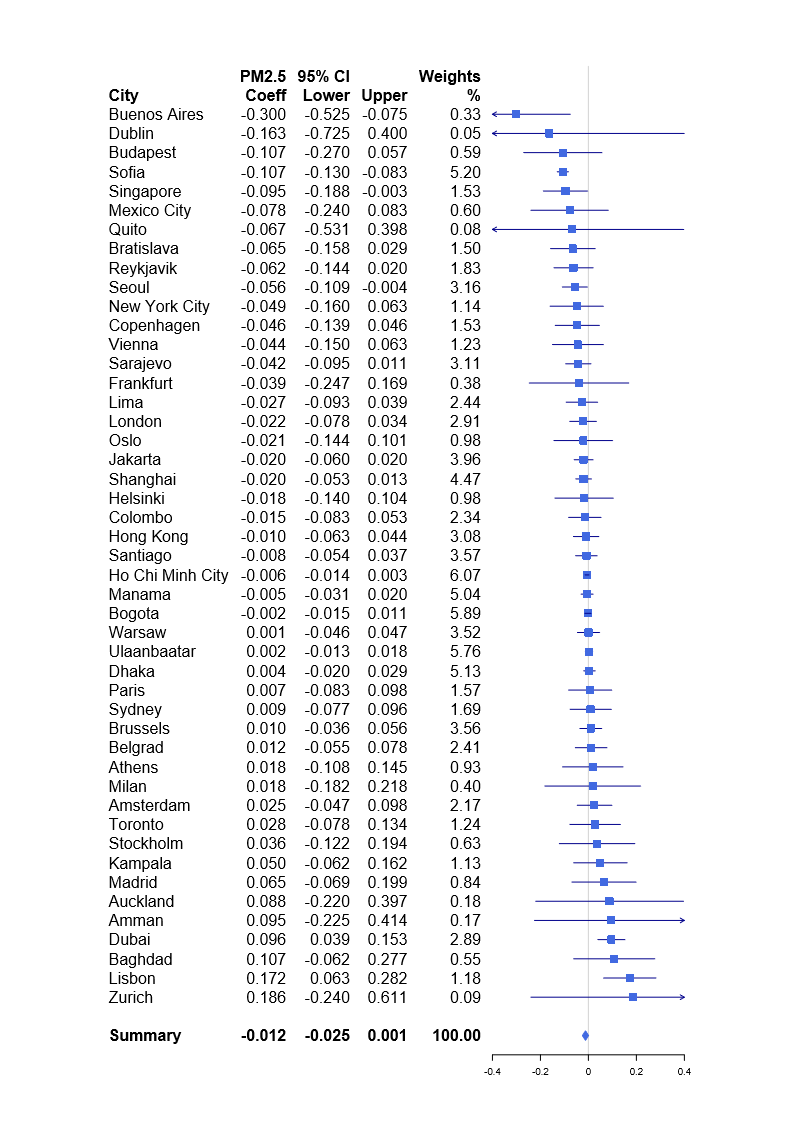


**A Figure 6.** Regression coefficients and confidence intervals for the association between same day PM2.5 concentration and stock index returns. Stratified for time period of 2001-2005.


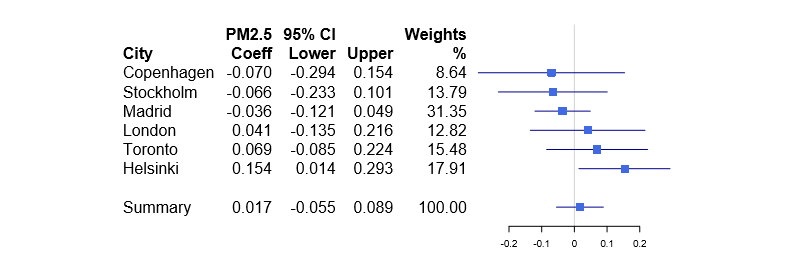


**A Figure 7.** Regression coefficients and confidence intervals for the association between same day PM2.5 concentration and stock index returns. Stratified for time period of 2006-2010.


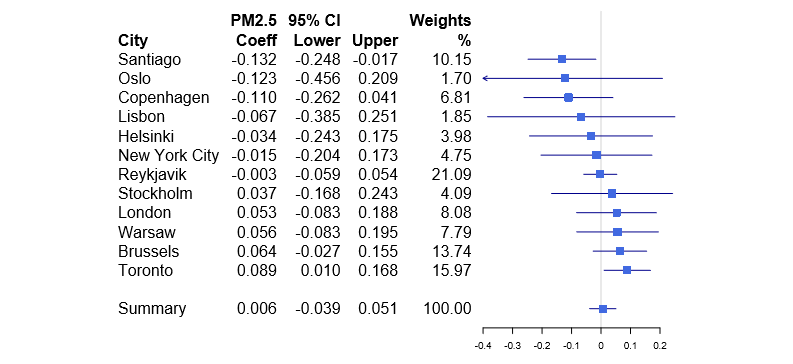


**A Figure 8.** Regression coefficients and confidence intervals for the association between same day PM2.5 concentration and stock index returns. Stratified for time period of 2011-2015.


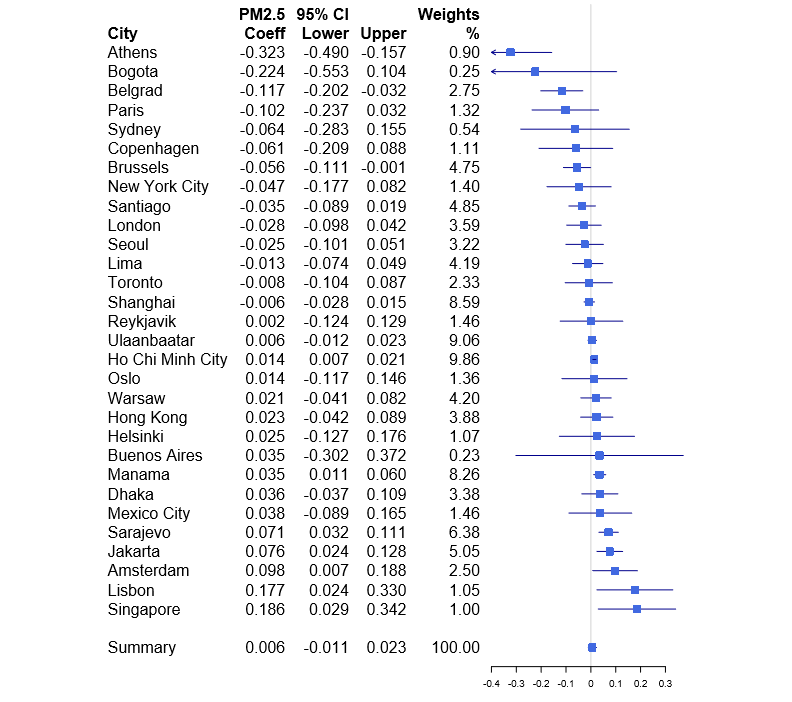


**A Figure 9.** Regression coefficients and confidence intervals for the association between same day PM2.5 concentration and stock index returns. Stratified for time period of 2016-2019
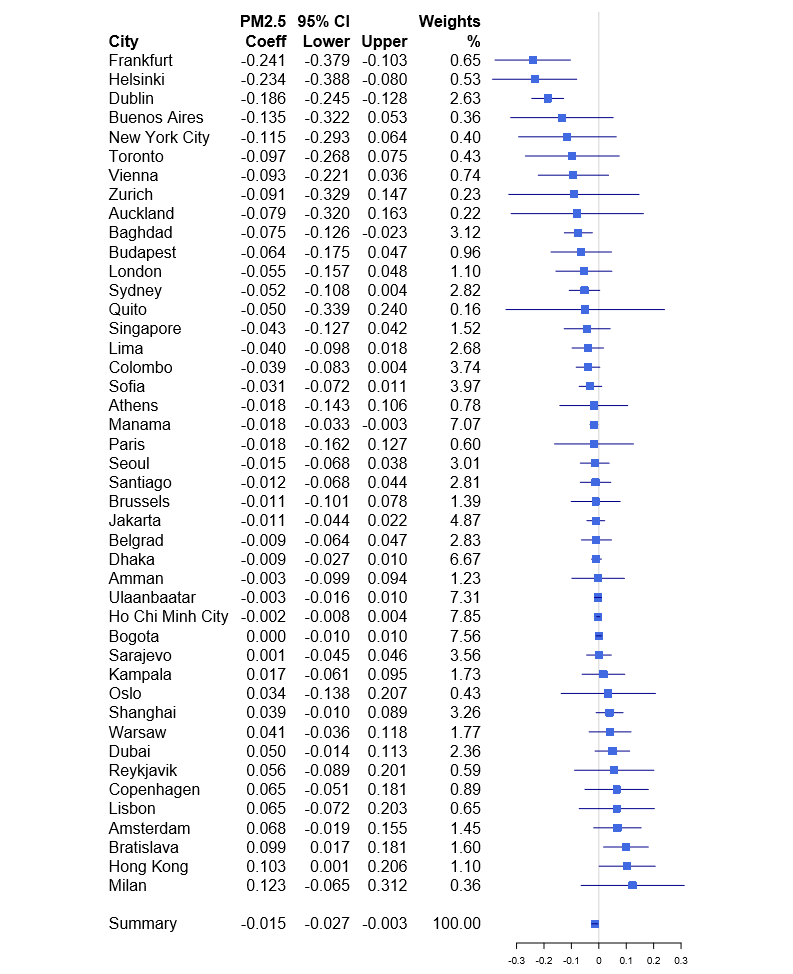


**A Figure 10.** Regression coefficients and confidence intervals for the association between same day PM2.5 concentration and stock index returns. Subset of Europe.
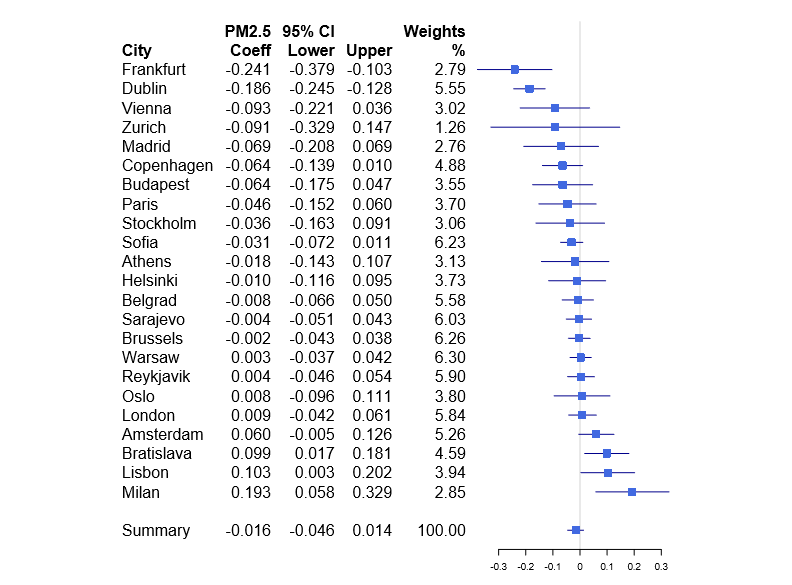


.

**A Figure 11.** Regression coefficients and confidence intervals for the association between same day PM2.5 concentration and stock index returns. Subset of Europe adjusted for NO_2_.
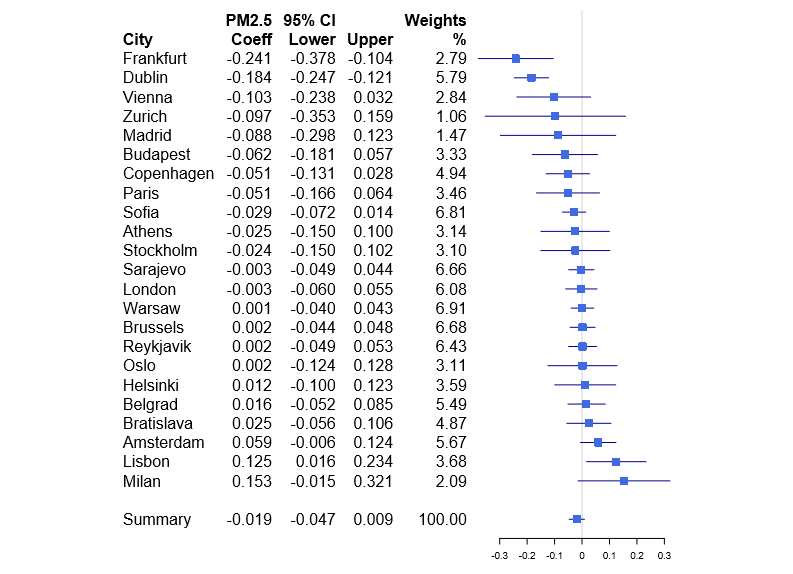


**A Figure 12.** Regression coefficients and confidence intervals for the association between same day PM2.5 concentration and stock index volatility. Subset of Europe.
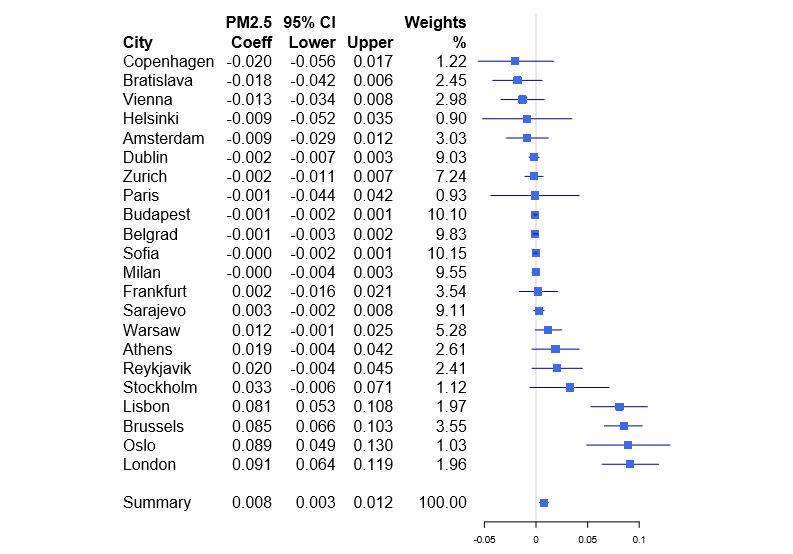


**A Figure 13.** Regression coefficients and confidence intervals for the association between same day PM2.5 concentration and stock index volatility. Subset of Europe adjusted for NO_2_.
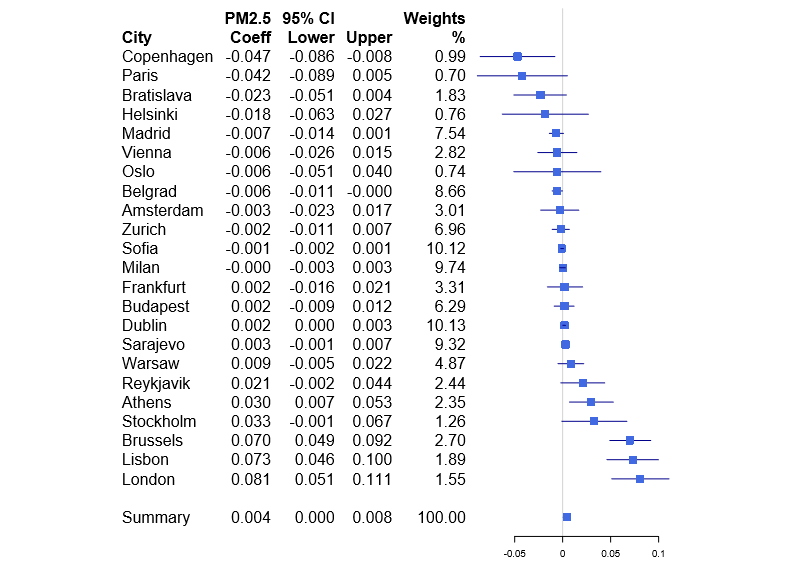


**A Table 1.** Data sources and distance from local stock exchange

| **Country** | **City** | **Stock Index** | **Latitude** | **Longitude** | **AQ Source** | **AQ Site ID** | **AQ distance (m)** | **GSOD ID** | **GSOD distance (m)** |
| --- | --- | --- | --- | --- | --- | --- | --- | --- | --- |
| **Argentina** | Buenos Aires | S&P Merval | -34.60 | -58.37 | ACUMAR |  | 6532 | 875820 | 6518 |
| **Australia** | Sydney | S&P/ASX 200 | -33.86 | 151.21 | NSW gov |  | 4142 | 947680 | 1690 |
| **Austria** | Vienna | ATX | 48.22 | 16.37 | EEA | at90tab | 1180 | 110340 | 1782 |
| **Bahrain** | Manama | Bahrain all-share | 26.24 | 50.57 | U.S. Department of State |  | 3672 | 411500 | 7093 |
| **Bangladesh** | Dhaka | DSE30 | 23.72 | 90.42 | U.S. Department of State |  | 8188 | 419230 | 7388 |
| **Belgium** | Brussels | BEL 20 | 50.85 | 4.35 | EEA | betr001 | 1182 | 64470 | 5343 |
| **Bosnia** | Sarajevo | Sarajevo10 | 43.86 | 18.41 | EEA | ba0041a | 50422 | 146540 | 2123 |
| **Botswana** | Gaborone | BSE Domestic | -24.68 | 25.93 |  |  |  | 682440 | 1725 |
| **Brazil** | Sao Paulo | Bovespa | -23.55 | -46.63 |  |  |  | 837790 | 4191 |
| **Bulgaria** | Sofia | BSESOFIX | 42.70 | 23.32 | EEA | bg0050a | 2354 | 156140 | 7299 |
| **Canada** | Toronto | S&P/TSX Composite Index | 43.65 | -79.38 | Ministry of the Environment, Conservation and Parks |  | 1681 | 715080 | 2478 |
| **Chile** | Santiago | SPCLXIPSA | -33.44 | -70.65 | SINCA |  | 1919 | 855770 | 3353 |
| **China** | Shanghai | SSE Composite Index | 31.14 | 121.30 | U.S. Department of State |  | 15116 | 588470 | 4134 |
| **China** | Shenzen | SZSE Component Index | 22.55 | 114.08 |  |  |  | 450320 | 6890 |
| **Colombia** | Bogota | COLCAP | 4.63 | -74.07 | U.S. Department of State |  | 3277 | 802220 | 11875 |
| **Croatia** | Zagreb | CROBEX | 45.80 | 15.97 |  |  |  | 142360 | 280 |
| **Cyprus** | Nikosia | Cyprus Main Market | 35.16 | 33.35 |  |  |  | 176070 | 4653 |
| **Czech Republic** | Prague | PX | 50.09 | 14.43 | EEA | cz0akal | 1221 | 115190 | 2546 |
| **Denmark** | Copenhagen | OMXC 20 | 55.68 | 12.58 | EEA | dk0034a | 754 | 61800 | 7847 |
| **Dubai** | Dubai | ADX General | 25.23 | 55.29 | U.S. Department of State |  | 4065 | 411940 | 8213 |
| **Ecuador** | Quito | Guayaquil Select | -0.20 | -78.49 | REMMAQ | Belisario | 2142 | 840720 | 4323 |
| **Egypt** | Cairo | EGX 30 | 30.05 | 31.24 |  |  |  | 623750 | 2326 |
| **Finland** | Helsinki | OMXH25 | 60.17 | 24.95 | EEA | fi00564 | 567 | 29750 | 10947 |
| **France** | Paris | CAC 40 | 48.89 | 2.25 | EEA | fr04002 | 5685 | 71560 | 10211 |
| **Germany** | Frankfurt | DAX Index | 50.14 | 8.57 | EEA | dehe135 | 6762 | 106370 | 12404 |
| **Greece** | Athens | Athens General Composite | 37.99 | 23.70 | EEA | gr0003a | 2153 | 167160 | 12416 |
| **Hong Kong** | Hong Kong | Hang Seng Index | 22.29 | 114.27 | Environmental Protection Department of HKSAR |  | 5905 | 450040 | 10769 |
| **Hungary** | Budapest | Budapest SE | 47.50 | 19.05 | EEA | hu0057a | 161075 | 128430 | 12527 |
| **Iceland** | Reykjavik | SE ICEX | 64.14 | -21.89 | EEA | is006a | 1023 | 41300 | 783 |
| **India** | Mumbay | BSE Sensex | 18.93 | 72.83 | OGD India |  |  | 430570 | 3720 |
| **Indonesia** | Jakarta | IDX Composite | -6.22 | 106.81 | U.S. Department of State | Jakarta South |  | 967450 | 5240 |
| **Iraq** | Baghdad | ISX Main 60 | 33.31 | 44.43 | U.S. Department of State |  | 2983 | 406500 | 18541 |
| **Ireland** | Dublin | ISEQ | 53.35 | -6.26 | EEA | ie0028a | 1475 | 39690 | 8458 |
| **Israeli** | Tel Aviv | TA35 | 32.06 | 34.77 |  |  |  | 401762 | 5986 |
| **Italy** | Milan | FTSE MIB | 45.46 | 9.18 | EEA | it1016a | 1318 | 160800 | 7627 |
| **Ivory Coast** | Abidjan | BRVM 10 | 5.32 | -4.02 |  |  |  |  |  |
| **Jamaica** | Kingston | JSE Market | 17.96 | -76.79 |  |  |  | 783970 | 3218 |
| **Japan** | Tokyo | Nikkei 225 | 35.68 | 139.78 |  |  |  | 476620 | 1067 |
| **Jordan** | Amman | Amman SE General | 31.98 | 35.91 | U.S. Department of State |  | 4499 | 402700 | 7894 |
| **Kazakhstan** | Almaty | KASE | 43.24 | 76.92 |  |  |  | 368700 | 1498 |
| **Kenya** | Nairobi | Kenya NSE 20 | -1.27 | 36.81 | OpenAQ | Alliance Girls High | 16443 | 637420 | 5720 |
| **Lebanon** | Beirut | BLOM Stock | 33.53 | 35.30 |  |  |  |  |  |
| **Malaysia** | Kuala Lumpur | KLCI | 3.15 | 101.70 |  |  |  | 486470 | 17047 |
| **Malta** | Valletta | MSE | 35.90 | 14.51 | EEA | mt00005 | 1950 | 165970 | 5222 |
| **Mauritius** | Port Louis | Semdex | -20.17 | 57.51 |  |  |  | 619950 | 15538 |
| **Mexico** | Mexico City | IPC Mexico | 19.43 | -99.16 | Instituto Nacional De Ecologia y Cambio Climátio |  |  | 766800 | 3791 |
| **Mongolia** | Ulaanbaatar | MNE Top 20 | 47.92 | 106.92 | U.S. Department of State |  | 1584 | 442920 | 13890 |
| **Montenegro** | Podgorica | MNSE 10 | 42.44 | 19.25 |  |  |  | 134624 | 3089 |
| **Morocco** | Casablanca | Moroccan All-Shares | 33.60 | -7.60 |  |  |  | 601550 | 6769 |
| **Namibia** | Windhoek | NSX | -22.58 | 17.09 |  |  |  | 681100 | 1658 |
| **Netherlands** | Amsterdam | AEX index | 52.37 | 4.90 | EEA | nl00017 | 1840 | 62400 | 11553 |
| **New Zealand** | Auckland | NZX50 | -36.84 | 174.77 | Auckland Council | Queen Street | 362 | 931120 | 13695 |
| **Nigeria** | Lagos | NSE30 | 6.45 | 3.39 |  |  |  | 652020 | 5294 |
| **Norway** | Oslo | OSEAX | 59.91 | 10.75 | EEA | no0073a | 1868 | 14920 | 4896 |
| **Oman** | Muscat | MSM30 | 23.60 | 58.55 |  |  |  | 412580 | 4569 |
| **Pakistan** | Karachi | Karachi 100 | 24.85 | 67.00 | U.S. Department of State |  | 1354 | 417810 | 7901 |
| **Palestine** | Nablus | AI Quds | 32.22 | 35.26 |  |  |  |  |  |
| **Peru** | Lima | SP Lima General | -12.05 | -77.03 | U.S. Department of State |  | 8941 | 846280 | 9461 |
| **Philippines** | Manila | PSEi Composite | 14.58 | 121.06 |  |  |  | 984300 | 7866 |
| **Poland** | Warsaw | WIG 20 | 52.23 | 21.02 | EEA | pl0140a | 1767 | 123750 | 8117 |
| **Portugal** | Lisbon | PSI 20 | 38.72 | -9.15 | EEA | pt03072 | 3087 | 85350 | 587 |
| **Qatar** | Doha | QE General | 25.33 | 51.53 |  |  |  | 411680 | 3495 |
| **Romania** | Bucharest | BET | 44.44 | 26.11 |  |  |  | 154220 | 2322 |
| **Russia** | Moscow | MOEX | 55.75 | 37.61 |  |  |  | 276120 | 8929 |
| **Rwanda** | Kigali | Rwanda All-Share | -1.94 | 30.06 |  |  |  | 643870 | 9289 |
| **Saudi Arabia** | Riyadh | Tadawul All-Share | 24.69 | 46.69 |  |  |  | 404380 | 4741 |
| **Serbia** | Belgrad | Belex 15 | 44.81 | 20.46 | EEA | rs0032a | 1172 | 132740 | 1313 |
| **Singapore** | Singapore | STI Index | 1.28 | 103.85 | NEA Singapore |  | 3835 | 486940 | 11294 |
| **Slovakia** | Bratislava | SAX | 48.15 | 17.11 | EEA | sk0004a | 519 | 118160 | 7941 |
| **Slovenia** | Ljubljana | Blue Chip SBITOP | 46.06 | 14.51 |  |  |  | 140150 | 1455 |
| **South Africa** | Johannesburg | South Africa Top 40 | -26.10 | 28.06 |  |  |  | 682672 | 7727 |
| **South Korea** | Seoul | KOSPI | 37.52 | 126.93 | AirKorea |  | 6282 | 471105 | 4513 |
| **Spain** | Madrid | IBEX35 | 40.42 | -3.69 | EEA | es0113a | 612 | 82220 | 763 |
| **Srilanka** | Colombo | CSE All-Share | 6.93 | 79.84 | U.S. Department of State |  | 2208 | 434660 | 4438 |
| **Sweden** | Stockholm | OMX 30 | 59.34 | 18.12 | EEA | se0050a | 2951 | 24840 | 3343 |
| **Switzerland** | Zurich | Swiss Market Index | 47.39 | 8.51 | EEA | ch0010a | 2371 | 66600 | 4644 |
| **Taiwan** | Taipei | Taiwan Weighted | 25.03 | 121.56 | EPA Taiwan |  | 3838 | 466960 | 4136 |
| **Tanzania** | Dar es Salaam | Tanzania All-Share | -6.81 | 39.29 |  |  |  | 638940 | 11923 |
| **Thailand** | Bangkok | SET Index | 13.76 | 100.57 | OpenAQ | Din Daeng | 1923 | 484550 | 3418 |
| **Tunisia** | Tunis | Tunindex | 36.85 | 10.27 |  |  |  | 607150 | 3752 |
| **Turkey** | Istanbul | BIST100 | 41.11 | 29.04 |  |  |  | 170610 | 3536 |
| **Uganda** | Kampala | Uganda All-Share | 0.33 | 32.62 | U.S. Department of State |  | 4450 | 636800 | 1263 |
| **Ukraine** | Kiev | PFTS | 50.44 | 30.50 |  |  |  | 333450 | 5265 |
| **United Kingdom** | London | FTSE 100 | 51.52 | -0.10 | EAA | gb0566a | 2027 | 37683 | 10729 |
| **United States** | New York City | S&P 500 | 40.71 | -74.01 | EPA US | 360610134 | 1594 | 725020 | 13573 |
| **Venezuela** | Caracas | Bursatil | 10.49 | -66.86 |  |  |  | 804160 | 1952 |
| **Vietnam** | Ho Chi Minh City | VN30 | 10.77 | 106.70 | U.S. Department of State |  | 1619 | 489000 | 7843 |
| **Zambia** | Lusaka | LSE All-Share | -15.43 | 28.31 |  |  |  | 676660 | 2323 |

**A Table 2.**  Volatility model with index day PM_2.5_ concentrations. Estimates for GJR-GARCH parameters and continuous predictors.

| City | omega | alpha1 | beta1 | gamma1 | pm25 | lag1_ret | lag2_ret | PRCP | WDSP | SLP | VISIB |
| --- | --- | --- | --- | --- | --- | --- | --- | --- | --- | --- | --- |
| Amman | 0.0000 | 0.0496 | 0.8972** | 0.0590 | -0.0013 | -0.0004 | -0.0023 | -0.0001 | -0.0036 | -0.0018 | -0.0006 |
| Amsterdam | 0.0159 | 0.0035 | 0.9286** | 0.0959 | -0.0177 | -0.0218* | -0.0226* | 0.0045* | -0.0014 | 0.0011 | 0.0017 |
| Athens | 0.0351* | 0.0301 | 0.9201** | 0.0503 | 0.0140 | -0.0091 | -0.0122 | -0.0045 | 0.0064 | 0.0026 | -0.0001 |
| Auckland | 0.0007 | 0.0004 | 0.9996** | -0.0044 | -0.0065* | 0.0018* | 0.0008 | -0.0001 | -0.0001 | 0.0002* | -0.0003* |
| Baghdad | 0.0001 | 0.0504 | 0.9022** | 0.0453 | 0.0009 | -0.0015 | 0.0003 | -0.0024* | 0.0044* | -0.0007 | 0.0027 |
| Belgrad | 0.0011 | 0.0161 | 0.9737** | 0.0055 | 0.0007 | 0.0023 | 0.0035 | -0.0000 | 0.0064** | 0.0012** | -0.0011** |
| Bogota | 0.0007 | 0.0564 | 0.9498** | -0.0201 | -0.0037* | -0.0005 | -0.0068 | -0.0004 | 0.0103 | -0.0011 | 0.0021 |
| Bratislava | 0.0007 | 0.0601 | 0.9072 | 0.0328 | -0.0149 | -0.0034 | -0.0053 | -0.0051 | -0.0060 | 0.0005 | 0.0011 |
| Brussels | 0.0026 | 0.0302 | 0.9346** | 0.0616** | 0.0857** | -0.0223** | -0.0400** | 0.0058* | 0.0263** | -0.0021 | -0.0039** |
| Budapest | 0.0009** | 0.0108 | 0.9994** | -0.0253 | -0.0005 | 0.0008* | 0.0006 | -0.0000 | -0.0001 | 0.0000 | -0.0000 |
| Buenos Aires | 0.0153 | 0.0323 | 0.9640** | 0.0036 | -0.0147 | -0.0100 | -0.0114 | 0.0006 | 0.0207 | -0.0016 | -0.0067 |
| Colombo | 0.0001 | 0.0534 | 0.8956** | 0.0648 | 0.0053 | -0.0107 | -0.0242** | 0.0001 | 0.0105 | 0.0066 | 0.0010 |
| Copenhagen | 0.0422 | 0.0592 | 0.8551** | 0.1563 | -0.0210 | -0.0604** | -0.0610** | -0.0014 | -0.0017 | 0.0004 | -0.0052 |
| Dhaka | 0.0003 | 0.0613 | 0.9016** | 0.0442 | 0.0029 | -0.0249* | -0.0093 | -0.0003 | 0.0832** | 0.0110** | -0.0518** |
| Dubai | 0.0007 | 0.0332 | 0.9689** | -0.0096 | 0.0019 | -0.0029 | -0.0001 | -0.0034* | 0.0016 | -0.0026 | 0.0080 |
| Dublin | 0.0007 | 0.0232 | 0.9691** | 0.0098 | -0.0111 | -0.0024 | -0.0017 | 0.0012 | -0.0043 | -0.0004 | -0.0011 |
| Frankfurt | 0.0017 | 0.0052 | 0.9752** | 0.0327 | 0.0025 | -0.0083 | -0.0096 | 0.0015 | -0.0007 | -0.0009 | 0.0015 |
| Helsinki | 0.0028 | 0.0475 | 0.9172** | 0.0605** | -0.0095 | -0.0406** | -0.0482** | -0.0028 | -0.0203* | -0.0035** | -0.0010 |
| Ho Chi Minh City | 0.0015 | 0.0379** | 0.9715** | -0.0215 | -0.0019 | -0.0056 | -0.0090 | -0.0001 | 0.0294** | 0.0026 | -0.0239 |
| Hong Kong | 0.0014 | 0.0468 | 0.9426** | 0.0089 | 0.0019 | -0.0102 | -0.0105 | -0.0003 | -0.0050 | -0.0007 | 0.0110** |
| Jakarta | 0.0006 | 0.0547 | 0.9069** | 0.0399 | 0.0129** | -0.0060 | -0.0118 | -0.0002 | -0.0083 | -0.0035 | 0.0296 |
| Kampala | 0.0012 | 0.0134 | 0.9992** | -0.0291 | -0.0106 | -0.0088 | -0.0158 | -0.0001 | 0.0040 | 0.0007 | -0.0157* |
| Lima | 0.0007 | 0.0318* | 0.9621** | 0.0086 | 0.0175** | 0.0215** | 0.0249** | -0.0078 | -0.0106 | 0.0137** | -0.0271** |
| Lisbon | 0.0647* | 0.0369 | 0.8450** | 0.1402** | 0.0769** | -0.0077 | -0.0083 | 0.0045** | 0.0563** | 0.0080** | 0.0025** |
| London | 0.0018 | 0.0422* | 0.9133** | 0.0752** | 0.0902** | -0.0127 | -0.0238** | -0.0018 | -0.0051 | -0.0019 | 0.0602** |
| Madrid | 0.0004 | 0.0598 | 0.9208** | 0.0204 | -0.0086 | -0.0029 | 0.0056 | -0.0006 | -0.0062 | 0.0004 | -0.0035 |
| Manama | 0.0004 | 0.0279 | 0.9610** | 0.0171 | 0.0018* | -0.0064 | -0.0030 | -0.0036* | 0.0013 | 0.0004 | -0.0001 |
| Mexico City | 0.0013 | 0.0104 | 0.9981** | -0.0214 | 0.0043 | -0.0254** | -0.0182** | 0.0007 | 0.0078 | -0.0087** | -0.0024 |
| Milan | 0.0020 | 0.0010 | 0.9993** | -0.0075 | 0.0002 | -0.0000 | -0.0001 | 0.0000 | -0.0003 | -0.0000 | 0.0000 |
| New York City | 0.0017 | 0.0519 | 0.9095** | 0.0589 | 0.2052** | -0.0399** | -0.0357** | 0.0026 | 0.0147 | 0.0004 | -0.0154* |
| Oslo | 0.0025 | 0.0158 | 0.9442** | 0.0729** | 0.0948** | -0.0159* | -0.0139 | 0.0007 | 0.0010 | -0.0031** | 0.0042** |
| Paris | 0.0117 | 0.0126 | 0.9203** | 0.1272** | -0.0025 | -0.0277** | -0.0277** | -0.0001 | 0.0011 | -0.0057** | -0.0029 |
| Quito | 0.0001 | 0.0474 | 0.9328 | 0.0157 | -0.0201 | 0.0097 | 0.0060 | 0.0004 | 0.0099 | -0.0006 | -0.0049** |
| Reykjavik | 0.0025 | 0.0323 | 0.9517** | 0.0292 | 0.0066 | -0.0035 | -0.0025 | NA | 0.0020 | -0.0006 | -0.0014* |
| Santiago | 0.0008 | 0.0585** | 0.9095** | 0.0479* | -0.0153* | -0.0114 | -0.0210** | -0.0039 | 0.0680** | 0.0018 | 0.0043 |
| Sarajevo | 0.0022 | 0.0008** | 0.9996** | -0.0114 | 0.0012** | 0.0008 | 0.0004 | 0.0000 | -0.0006 | 0.0004** | -0.0000 |
| Seoul | 0.0008 | 0.0469 | 0.9324** | 0.0319 | -0.0194** | -0.0204** | -0.0274** | -0.0010 | -0.0290** | -0.0013 | 0.0017 |
| Shanghai | 0.0064 | 0.0593** | 0.9469** | -0.0170 | 0.0001 | 0.0025 | 0.0018 | -0.0015 | 0.0005 | 0.0207** | -0.0218** |
| Singapore | 0.0015 | 0.0188 | 0.9727** | 0.0104 | 0.0196** | -0.0044 | -0.0047 | 0.0005 | 0.0214** | 0.0023 | 0.0288** |
| Sofia | 0.0001 | 0.0450** | 0.9881** | -0.0766** | -0.0003 | 0.0030 | 0.0024 | -0.0001 | 0.0009 | -0.0000 | -0.0011 |
| Stockholm | 0.0197 | 0.0592 | 0.8730 | 0.1237 | 0.0290 | -0.0252** | -0.0167 | NA | 0.0030 | 0.0009 | -0.0040 |
| Sydney | 0.0007 | 0.0336 | 0.9454** | 0.0296 | -0.0163* | -0.0031 | -0.0148* | 0.0003 | -0.0556** | -0.0004 | -0.0005 |
| Toronto | 0.0086* | 0.0247* | 0.9256** | 0.0732** | -0.0261 | -0.0503** | -0.0476** | -0.0011 | 0.0653** | 0.0041* | -0.0021* |
| Ulaanbaatar | 0.0018 | 0.0197** | 0.9909** | -0.0269** | -0.0038** | 0.0076* | 0.0043 | -0.0019 | -0.0093* | 0.0010 | -0.0012 |
| Warsaw | 0.0013 | 0.0509 | 0.8995** | 0.0575 | 0.0096 | -0.0148* | -0.0214** | -0.0031 | -0.0024 | -0.0001 | 0.0053** |
| Vienna | 0.0012 | 0.0340 | 0.9303** | 0.0645 | -0.0144 | -0.0373** | -0.0331** | 0.0010 | 0.0038 | 0.0007 | -0.0007 |
| Zurich | 0.0007 | 0.0179 | 0.9556** | 0.0489 | -0.0019 | -0.0049 | -0.0042 | 0.0001 | 0.0012 | -0.0003 | 0.0003 |

Statistical significance denoted as: * < 0.05 , ** < 0.01
